# Prevalence of cardiovascular risk factors across six African Immigrant Groups in Minnesota

**DOI:** 10.1186/s12889-015-1740-3

**Published:** 2015-04-22

**Authors:** Barrett Sewali, Nonyelum Harcourt, Susan A Everson-Rose, Robert E Leduc, Sirad Osman, Michele L Allen, Kolawole S Okuyemi

**Affiliations:** Program in Health Disparities Research, University of Minnesota Medical School, Minneapolis, MN 55414 USA; Department of Family Medicine and Community Health, University of Minnesota Medical School, 717 Delaware St. SE. Ste. 166, Minneapolis, MN 55414 USA; Department of Medicine, University of Minnesota Medical School, Minneapolis, MN 55414 USA; Division of Biostatistics, School of Public Health, University of Minnesota, Minneapolis, MN 55414 USA; New American Community Services, Minneapolis, MN 55104 USA; Masonic Cancer Center, University of Minnesota, Minneapolis, MN 55414 USA; Department of Medicine, Cardiology Division, University of Minnesota, Minneapolis, MN 55414 USA

**Keywords:** Immigrant, African, Cardiovascular, Risk factors

## Abstract

**Background:**

Although African immigrants represent a large and growing segment of the U.S. population, there are little or no data available on the prevalence of cardiovascular disease (CVD) risk factors among this diverse population. This study compared the prevalence of self-reported CVD risk factors and health behaviors and examined the associations between immigration related characteristics and CVD risk factors and health behaviors across six African immigrants groups.

**Methods:**

Data were from 996 African immigrants in the U.S., (37.9% Somalis; 26.8% Ethiopians; 14% Liberians; 8.5% Sudanese; 5.1% Kenyans and 7.8% others group) from a cross-sectional survey conducted in the Twin cities of Minnesota. Logistic regression models estimated the associations of demographic characteristics, and immigration-related factors (length of stay in the United states, English proficiency, income and health insurance) with prevalence of CVD risk factors (overweight/obese; hypertension and diabetes mellitus) and self-reported health behaviors (cigarette smoking, physical inactivity, conscious effort to exercise and eating a healthy diet).

**Results:**

We found a relatively low self-reported prevalence of diabetes, hypertension, and smoking. However, significant differences were noted by country of origin. Using Somalis as our referent country of origin group, we found that Liberians and Kenyans were more likely to report having diabetes or hypertension. On all measures of health behaviors, Liberians were more likely to engage in more health protective behaviors than other individuals.

**Conclusions:**

Although African immigrants have different prevalence rates for CVD risk factors and health behaviors, there is a need to further explore the differences observed by country of emigration.

## Background

Cardiovascular disease (CVD) continues to be the leading cause of death in the United States (U.S.) [1]. Despite tremendous progress made in identifying the risk factors and health behaviors associated with CVD in the dominant racial and ethnic groups in the U.S., very few studies have examined the prevalence of these risk factors and health behaviors among African immigrants. Since CVD risk factors are largely influenced by social, cultural and behavioral factors [2,3], it is important to explore how cardiovascular risk factors and health behaviors compare across one of the fastest growing immigrant populations in the U.S.; immigrants from Africa [4,5].

African immigrants are a diverse group; they differ from each other by country of origin, reasons for migration, primary languages spoken, health practices and beliefs, human capital, education status, and cultural background [6]. However, most national surveys or instruments available where race is recorded do not capture these distinctions. Currently it is the norm for African immigrants to report their race as ‘Black’ [7]. Grouping African immigrants together as blacks, foreign-born blacks, or African Americans may miss important behavioral and lifestyle variations within this population [8].

Prior studies have shown that cardiovascular disease risk factors among U.S. immigrant populations differ by immigration characteristics such as length of stay in the US, English proficiency, age at migration, cultural and religious beliefs, education background, and socioeconomic status [9-11]. However, these data are mostly in reference to Hispanic immigrants who make up the largest immigrant population in the U.S. Several studies that explored cardiovascular disease risk factors among African immigrants focused mostly on West African immigrants from Nigeria, Ghana and Cameroon and found a high prevalence of both hypertension and overweight/obesity [12-14].

In light of the growing number and diversity of African immigrants in the U.S., the existing literature does not comprehensively demonstrate which African immigrant groups are at an increased risk of CVD. To address this gap in knowledge, we conducted a cross-sectional study that determined 1) the comparative prevalence of CVD risk factors (overweight/obese; hypertension and diabetes mellitus) and lifestyle behaviors (cigarette smoking, days of physical activity, conscious effort to exercise and eat a healthy diet) and 2) the study looked at immigration related factors influenced the CVD risk factors and health behaviors across 6 African immigrant groups.

## Methods

Data are from a survey conducted by New Americans Community Services (NACS), a local community service agency that serves African immigrants, refugees and asylees, from 2006 to 2007 in the Twin Cities area, focusing on neighborhoods with a high concentration of African immigrant families. Eligible participants self-identified as African immigrant adults, 18 years or older. The survey assessed demographic, social and economic factors, health care access and utilization, lifestyles and risk behaviors, and English language proficiency. Prior to conducting the survey, NACS facilitated focus groups to assist in the development of survey questions that identified health conditions and health care-seeking behaviors of African immigrants. The survey was conducted in-person at the participants’ homes in either English or participants’ preferred language. A total of 1600 households were approached, using 25 NACS employees that were trained in interview methodology. Informed consent was obtained from the individuals that agreed to participate in the study (a response rate of 63%). A total of 1009 participants completed the survey; 13 participants were excluded; 7 were born in the U.S., and 6 additional individuals were missing country of origin data, resulting in 996 individuals for the analytic sample. Survey protocol and all procedures related to the survey were reviewed and approved by the NACS community advisory board and the University of Minnesota Institutional Review Board.

### Assessments

#### Immigration-related factors and socio-demographic characteristics

The survey captured 18 different African countries of origin. Due to sample size constraints and for ease of interpretation we collapsed these countries into 6 categories: (1) Somalia, (2) Ethiopia, (3) Liberia, (4) Sudan, (5) Kenya, and (6) other countries. About 65% of our study participants were from the East African region specifically from Somalia and Ethiopia. Number of years in the U.S. was assessed based on their response to the question “When did you come to live in the U.S.?” Responses were dichotomized as ≥5 years versus < 5 years. Ability to understand English well was assessed from the question in the survey “How well do you understand English?” Respondents who indicated “not at all” or “not well” were classified as less than proficient, compared to those who responded “well” or “very well”, who were classified as proficient in English.

Age and sex were self-reported. Access to care was assessed using health insurance status, reported as no insurance, private insurance, or public insurance. Private insurance was defined as health insurance obtained by participants as a result of their work place, while public insurance was defined as one that participants obtained from the state given their immigration and or economic status. Difficulty meeting household expenses, a proxy for household income, was assessed from the question: “During the past 12 months, how often did it happen that you did not have enough money to meet your family’s expenses, such as bills, food, clothes, or other things your household needed?” Response options were never, rarely, sometimes and often. This variable was dichotomized as never/rarely and sometimes/often for analysis.

#### Cardiovascular risk factors

CVD-related risk factors were prevalence of overweight/obese, defined by body mass index (BMI), calculated as weight in kilograms divided by height in meters-squared, based on self-reported height and weight. BMI ≥ 25 was considered overweight/obese with BMI < 25 as the referent. Diabetes or hypertension prevalence was assessed based on self-reported information about whether the respondent had ever been told by a doctor or health professional that he or she had that condition (yes or no) or whether they are currently controlling the condition by medication, exercise or diet. For analysis purposes we excluded women who reported that they were told by their doctor or healthcare provider that they had either condition only during pregnancy.

#### Health behaviors

Four CVD-related health behaviors were assessed:

- *Smoking status:* This was categorized as never or former smoker versus current smoker, defined as someone who currently smoked and had smoked at least 100 cigarettes in his/her lifetime.
- *Physical Activity:* This was assessed using responses from the question “During an average week, how many days do you get at least 30 minutes of moderate physical activity?” We coded physical activity as ≥ 5 days per week versus <5 days.
- *Conscious effort to exercise:* This was assessed from responses to the question “To what extent do you make a conscious effort to regularly get exercise and physical activity?” For ease of interpretation the variable was categorized into high level of effort versus low level of effort to engage in regular exercise.
- *Conscious effort to achieve a healthy diet:* This was assessed using the question “To what extent do you carefully select what you eat to achieve a healthy diet?” The variable was categorized as very careful/somewhat careful and not careful at all.

### Statistical analysis

Demographic characteristics were summarized as means and standard deviations for continuous variables and number occurring and percent for discrete variables. Proportions were compared univariately across national origin groups by Pearson's Chi-square test. Logistic regression was used to estimate the odds ratio for self-reported CVD-related health behaviors and presence of CVD risk factors by national origin relative to Somali’s as our referent group. Models were adjusted for sex, age, length of stay in the US, self-reported English proficiency, self-reported difficulty in meeting household expenses, and self-reported insurance status. These factors were pre-specified prior to analysis. For categorical variables with more than 2 levels, p-values are reported for the test of association of the overall variable with the outcome as well as for specific levels of the variable against the referent group.

## Results

Table 1 shows demographic characteristics of our overall cohort and by country of origin. The mean age was 35 years (S.D., 13.3), slightly more than half of the participants were males, more than three quarters had been in the U.S for ≥ 5 years, 77.3% understood English well, and 60% indicated they did not have trouble meeting their daily expenses, while 94.6% had public or private health insurance. Somalis comprised of the largest group with 38.7%; followed by Ethiopians 26.8%; Liberians 14%; Sudanese 8.5%; Kenyans 5.1% and 7.8% were categorized as other countries.

**Table 1:** **Demographic characteristics of participants**

			**Country of origin groups**					
**Characteristic**		**Overall cohort**	**Somali**	**Ethiopia**	**Liberia**	**Sudan**	**Kenya**	**Others** ^**1**^
Females		470 (47.8)	211 (56)	121 (45.3)	58 (41.7)	34 (40.5)	20 (39.2)	26 (33.3)
Males		526 (52.8)	166 (44)	146 (54.7)	81 (58.3)	50 (59.5)	31 (60.8)	52 (66.7)
Age^2^		35.1 (13.3)	35 (13.9)	37.1 (14.3)	33.2 (13.7)	34.5 (8.7)	35.1 (13.2)	32.7 (9.9)
Length of Stay ≥5 Years		758 (76.2)	257 (68.2)	205 (76.8)	118 (84.9)	73 (88)	39 (76.5)	66 (84.6)
Understand English Well		765 (77.3)	232 (62.2)	202 (76.2)	138 (99.3)	73 (86.9)	49 (96.1)	71 (91)
Trouble Meeting Expenses (Never or Rarely)		590 (60)	278 (74.9)	196 (74.8)	31 (22.5)	37 (44)	22 (43.1)	26 (33.3)
Insurance Status	No Insurance	54 (5.4)	16 (4.3)	18 (6.8)	4 (2.9)	10 (11.9)	1 (2)	5 (6.4)
	Private Insurance	543 (54.7)	129 (34.4)	149 (56)	123 (88.5)	38 (45.2)	43 (84.3)	61 (78.2)
	Public Insurance	396 (39.9)	230 (61.3)	99 (37.2)	12 (8.6)	36 (42.9)	7 (13.7)	12 (15.4)
Total sample		996 (100)	377 (37.9)	267 (26.8)	139 (14)	84 (8.5)	51 (5.1)	78 (7.8)

^1^Other as a country of origin group represented individuals from (Angola, Burundi, Cameroon, Congo/Zaire, Ghana, Eritrea, Sierra Leon, South Africa, Tanzania, Togo and Uganda). This was done due to the small participant representation from each of these countries.

^2^Age is mean (SD) and all other values shown are n (%).

Table 2 shows the overall prevalence of CVD risk factors and healthy behaviors by country of origin. Furthermore, the CVD risk factors by country of origin were statistically different. Besides never smoking all other healthy behaviors were statistically different across the six country groups. Fifty-five percent of the participants were classified as being obese or overweight. There is a significant difference in hypertension across national origin groups. The overall prevalence is 8%, with prevalence just under 16% among Liberians and Kenyans and as low as 4% among Sudanese. More than ninety percent reported they never smoked cigarettes, 28% reported engaging in at least 5 days of moderate activity, 58.6% made a conscious effort to exercise, while 78.7% reported making a conscious effort to eat a healthy diet.

**Table 2:** **Prevalence of CVD risk factors and healthy behaviors by Country of origin group**

	**Somali**		**Ethiopia**		**Liberia**		**Sudan**		**Kenya**		**Other**			**Overall**	
**Characteristic**	**N**	**%**	**N**	**%**	**N**	**%**	**N**	**%**	**N**	**%**	**N**	**%**	**P-value**	**N**	**%**
**CVD risk factors**															
Overweight/Obese	185	49.5	160	59.9	103	74.1	23	27.4	29	56.9	46	59.0	<.0001	546	55.0
Diabetes	16	4.5	19	7.1	7	5.1	4	4.8	4	7.8	3	3.9	0.6758	53	5.4
Hypertension	22	5.9	21	7.9	22	15.8	3	3.7	8	15.7	6	7.7	0.0030	82	8.3
**Healthy behaviors**															
Never Smoked	346	92.0	238	89.8	131	96.3	74	88.1	49	96.1	69	89.6	0.1414	907	91.7
At least 5 days moderate activity	87	26.7	62	24.7	59	43.7	19	25.0	15	30.6	13	17.8	0.0004	255	28.0
Make a conscious effort to exercise	188	50.3	133	51.0	119	86.2	36	43.4	39	76.5	62	79.5	<.0001	577	58.6
Make a conscious effort to eat a healthy diet	287	77.6	209	80.1	130	93.5	46	55.4	42	82.4	59	75.6	<.0001	773	78.7
Total	377	100.0	267	100.0	139	100.0	84	100.0	51	100.0	78	100.0		996	100.0

Table 3 shows results from logistic regression models evaluating odds of CVD risk factors by country of origin, demographics, immigration related and socio-economic factors. Liberians were more likely to be overweight or obese compared to Somalis (p < .0001), and were nearly four times more likely to report being told that they were either diabetic or hypertensive (p = 0.018). Sudanese were less likely to be overweight or obese (p < .0001), while Kenyans were more likely to be told they were diabetic or hypertensive compared to Somalis (p = 0.032). Several covariates were significantly related to CVD risk factor prevalence, including age, difficulty meeting expenses, length of stay in the U.S. and health insurance status. Specifically, increasing age was associated with greater odds of reporting diabetes or hypertension (p < .001), while those individuals who never or rarely had difficulty meeting family expenses were less likely to report either diabetes or hypertension (p < .003) after adjustment for other factors. Individuals that reported staying in the U.S. ≥ 5 years were more likely to be classified as overweight or obese (p = 0.029), while individuals that had no health insurance were less likely to be classified as overweight or obese (p < .004).

**Table 3:** **Logistic regression models for odds of** ***CVD Risk Factors***

		**Overweight/Obese**		**Diabetes or hypertension**	
**Country**		**OR**	**95% CI**	**OR**	**95% CI**
Group	Somalia	**referent**			
	Ethiopia	1.45	[1.03,2.04]	1.58	[0.82,3.08]
	Kenya	1.16	[0.62,2.18]	4.42	[1.65,11.83]
	Liberia	2.67	[1.61,4.4]	3.83	[1.69,8.67]
	Sudanese	0.39	[0.23,0.68]	1.3	[0.45,3.8]
	Others	1.33	[0.77,2.27]	2.07	[0.76,5.63]
**Characteristic**		**OR**	**95% CI**	**OR**	**95% CI**
Female		1.15	[0.87,1.51]	0.82	[0.5,1.35]
Age		1.01	[1,1.02]	1.09	[1.07,1.11]
Length of Stay	≥ 5 years	1.47	[1.04,2.08]	0.99	[0.55,1.78]
Understand English Well		0.73	[0.48,1.1]	0.89	[0.44,1.79]
Trouble Meeting Expenses	Never or Rarely	1.1	[0.81,1.49]	0.45	[0.27,0.77]
Insurance Status	Private insurance	**referent**			
	No insurance	0.31	[0.17,0.59]	0.14	[0.01,2.41]
	Public insurance	0.62	[0.44,0.87]	1.23	[0.65,2.35]

Table 4 shows correlates of health behaviors (not smoking, physical activity, conscious effort to exercise and eat a healthy diet). There was no significant difference in self-reported smoking by country of origin. Liberians had 48% higher odds of reporting 5 days or more of physical activity (p = 0.001). Liberians were more likely to make a conscious effort to exercise (p < .005), while Sudanese were less likely to report making a conscious effort to exercise. Liberians had higher odds of making a conscious effort to eat a healthy diet (p = 0.001), while individuals from Sudan were less likely to report making a conscious effort to eat a healthy diet (p < .001). Significant covariates in the analyses of health behaviors included sex, understanding English, trouble meeting expenses, and age. Females were four times more likely than males to report never smoking (p = 0.001) after adjusting for other covariates in the model. Individuals that reported understanding English well were 3 times more likely to report making a conscious effort to exercise (p < .001), while those individuals that never or rarely had trouble meeting expenses were less likely to report making a conscious effort to exercise. Individuals that were older and reported understanding English well were more likely to report making a conscious effort to eat a healthy diet (p < .005) after adjustment for other factors.

**Table 4:** **Logistic regression models for odds of** ***Healthy Behaviors***

		**Never smoker**		**Physical activity**		**Conscious effort to exercise**		**Effort to eat a healthy diet**	
**Country**		**OR**	**95% CI**	**OR**	**95% CI**	**OR**	**95% CI**	**OR**	**95% CI**
Group	Somalia	**Referent**							
	Ethiopia	0.91	[0.5,1.64]	0.8	[0.54,1.2]	0.9	[0.65,1.29]	0.9	[0.58,1.37]
	Kenya	3.3	[0.73,14.96]	0.9	[0.44,1.77]	2.2	[1.06,4.41]	0.9	[0.38,1.97]
	Liberia	3.59	[1.26,10.23]	1.5	[0.9,2.44]	3.7	[2.09,6.63]	2.8	[1.27,6.25]
	Sudanese	0.87	[0.39,1.95]	0.8	[0.42,1.42]	0.6	[0.35,0.96]	0.3	[0.15,0.45]
	Others	1.2	[0.49,2.96]	0.4	[0.22,0.87]	2.7	[1.47,5.1]	0.7	[0.35,1.27]
**Characteristic**		**OR**	**95% CI**	**OR**	**95% CI**	**OR**	**95% CI**	**OR**	**95% CI**
Female		4.02	[2.25,7.2]	1.3	[0.92,1.71]	1.1	[0.82,1.45]	1.2	[0.85,1.72]
Age		0.99	[0.97,1.01]	1	[0.99,1.02]	1	[0.99,1.01]	1	[1.01,1.04]
Length of Stay	≥5 years	1.12	[0.6,2.1]	0.8	[0.54,1.21]	0.9	[0.64,1.34]	1.1	[0.69,1.61]
Understand English Well		0.74	[0.35,1.57]	1.1	[0.67,1.8]	2.8	[1.86,4.34]	3.8	[2.33,6.28]
Trouble Meeting Expenses	Never or Rarely	1.54	[0.92,2.58]	0.8	[0.55,1.08]	0.7	[0.51,0.96]	1.4	[0.99,2.06]
Insurance Status	Private insurance	**Referent**							
	No insurance	1.12	[0.41,3.06]	0.8	[0.43,1.67]	0.9	[0.49,1.65]	1.1	[0.51,2.28]
	Public insurance	0.97	[0.54,1.74]	0.6	[0.39,0.86]	1.1	[0.75,1.48]	1	[0.65,1.51]

## Discussion

This study compares the prevalence of CVD risk factors and protective health behaviors across six diverse African immigrant groups. Overall we found that East African immigrants in our study had a low prevalence of cardiovascular risk factors (hypertension or diabetes) after adjustment for other factors. These results are consistent with some prior studies showing that African immigrants have a low prevalence of chronic diseases such as hypertension and diabetes compared to U.S.-born whites [8,15-18]. However, several other studies have shown different results. Commodore-Mensah et al. found hypertension prevalence of 53% among Ghanaian & Nigerians (West Africans) in the Washington area in the U.S. [12]. Goosen et al. showed a higher prevalence of diabetes in Somali immigrants in the Netherlands compared to other populations [19]. These variations in cardiovascular risk factors among immigrants from different locations and different immigration histories warrants further exploration.

More than 50 percent of the individuals in this study were overweight or obese. This is consistent with some studies that have shown comparable overweight/obese rates in Somali immigrants in Norway [20], and data from National Health Interview Survey done in the U.S., showing an overweight/obese prevalence rate of 58% among African immigrants in the U.S. [21].

The prevalence of health behaviors (never smoking, making a conscious effort to exercise and eat a healthy diet), was above 50 percent while the prevalence of making an effort to engage in physical activity was low. The prevalence of cigarette smoking in our sample was very low, with 92% reporting that they were nonsmokers. The low smoking prevalence rates are consistent with other studies that showed low smoking rates in immigrants compared to U.S. born individuals [22].

Less than one third of the participants reported making a conscious effort to engage in 5 or more days of physical activity. Our findings are consistent with one study that showed that immigrants were less likely than US-born individuals to report discussing diet and exercise with clinicians [10]. The reported low prevalence rates of engaging in physical activities are comparable to the physical activity rates reported for U.S born individuals [23,24].

On further analysis we found significant differences in both CVD risk factors and health behaviors by country of origin. Liberians were more likely to engage in at least 5 days of physical activity in a week, make a conscious effort to exercise, and eat a healthy diet. However, Liberians were more likely to report being diabetic or hypertensive and were more likely to be overweight or obese compared to Somalis. A plausible explanation for this may be that African immigrants from the western part of Africa may have different dietary habits compared to individuals from the eastern part of Africa where Somalia is located. Another possible explanation is that Liberians may have been more conscious of their weight and thus were more likely to report engaging in exercise and physical activity compared to the Somalis. Ethiopians were not significantly different from the Somalis on all measures after adjusting for all covariates. This result may hold true considering that Ethiopia and Somali are both located in the eastern part of Africa and share a common border. Kenyans on the other hand were more likely to report having diabetes or hypertension.

Sudanese were less likely to report a conscious effort to exercise, less likely to report a conscious effort to eat a healthy diet and were less likely to report being overweight or obese. Our findings are consistent with a study done in Sudan that showed that the prevalence rates of engaging in physical activity are low in this population [25].

The diverse cultural practices for these groups may account for the observed differences in CVD risk factors and health behaviors. We observed that respondents who understood English language well and those who did not experience financial strain were more likely to report a conscious effort to exercise and make a conscious effort to eat a healthy diet. This may point to language as a barrier for immigrants accessing freely available healthcare messages. We conclude that those immigrants who understood English well are more likely to engage in protective health behaviors compared to African immigrants who do not understand English well, as many health messages are mostly in English. This is consistent with previous studies that suggest that low English proficiency is a barrier to healthcare utilization among immigrants [26,27].

Individuals with no health insurance were less likely to be classified as being overweight or obese. However, those that had some form of health insurance were more likely to report engaging in at least 5 days of physical activity per week. A plausible explanation is that individuals with health insurance are in more contact with the healthcare system and are thus advised to engage in physical activity.

We also observed differences based on socioeconomic status (defined as ability to meet daily expenses), where individuals that reported not having trouble meeting their daily expenses were more likely to report having been told they had either diabetes or hypertension and were more likely to report making a conscious effort to exercise. One possible explanation for this is that those individuals that are able to meet their daily expenses are more likely to visit a healthcare provider and are more likely to get regular health assessments.

As with any study, there are limitations that apply to this study that are worth mentioning. First, this is a secondary data analysis from a cross-sectional study; inferences cannot be made about causal associations. Second, CVD risk factors and health behaviors were based on self-report and self-declared health status, increasing the potential for bias. Finally, sample size and the restriction of the data to the Twin Cities area in Minnesota limits the generalizability of the results.

However, our study adds to the literature by providing an analysis of the prevalence of CVD risk factors and health behaviors among a diverse group of African immigrants, not only by proxy measures of acculturation but also by age, sex and other socioeconomic factors and, importantly, by country of origin.

The implication of this study is that though African immigrants have different prevalence rates for CVD risk factors and health behaviors across different locations, there is a need to further explore the differences observed. It is therefore crucial to identify which country immigrants originate from in order to appropriately assess CVD risk factors and health behaviors. Our results suggest that targeted interventions focusing on specific CVD risk profiles within specific immigrant populations by country of origin are warranted.

## Conclusion

Given the significant differences in the CVD risk factors and health behaviors observed among African Immigrants in the U.S., we suggest a closer examination of the prevalence of these risk factors, health behaviors and migration histories across an even wider immigrant community. This will help design culturally tailored and specific interventions for the unique needs of these diverse immigrant populations.
